# The Impact of Dietary and Metabolic Risk Factors on Cardiovascular Diseases and Type 2 Diabetes Mortality in Brazil

**DOI:** 10.1371/journal.pone.0151503

**Published:** 2016-03-18

**Authors:** Marcia C. de Oliveira Otto, Ashkan Afshin, Renata Micha, Shahab Khatibzadeh, Saman Fahimi, Gitanjali Singh, Goodarz Danaei, Rosely Sichieri, Carlos A Monteiro, Maria L. C. Louzada, Majid Ezzati, Dariush Mozaffarian, Global Burden of Diseases, Injuries, and Risk Factors Metabolic Risk Factors of Chronic Diseases Expert Group and Nutrition and Chronic Diseases Expert Group (NutriCoDE)

**Affiliations:** 1 Division of Epidemiology, Human Genetics and Environmental Sciences, the University of Texas Health Science Center, School of Public Health, Houston, Texas, United States of America; 2 Friedman School of Nutrition Science & Policy, Tufts University, Boston, Massachusetts, United States of America; 3 Department of Global Health and Population, Harvard School of Public Health, Boston, Massachusetts, United States of America; 4 Department of Food Science and Human Nutrition, Agricultural University of Athens, Athens, Greece; 5 Departament of Epidemiology, University of the State of Rio de Janeiro, Rio de Janeiro, Rio de Janeiro, Brazil; 6 Department of Nutrition, School of Public Health, University of São Paulo, Sao Paulo, Sao Paulo, Brazil; 7 Faculty of Medicine, School of Public Health, Imperial College of London, London, United Kingdom; College of Tropical Agriculture and Human Resources, University of Hawaii, UNITED STATES

## Abstract

**Background:**

Trends in food availability and metabolic risk factors in Brazil suggest a shift toward unhealthy dietary patterns and increased cardiometabolic disease risk, yet little is known about the impact of dietary and metabolic risk factors on cardiometabolic mortality in Brazil.

**Methods:**

Based on data from Global Burden of Disease (GBD) Study, we used comparative risk assessment to estimate the burden of 11 dietary and 4 metabolic risk factors on mortality due to cardiovascular diseases and diabetes in Brazil in 2010. Information on national diets and metabolic risks were obtained from the Brazilian Household Budget Survey, the Food and Agriculture Organization database, and large observational studies including Brazilian adults. Relative risks for each risk factor were obtained from meta-analyses of randomized trials or prospective cohort studies; and disease-specific mortality from the GBD 2010 database. We quantified uncertainty using probabilistic simulation analyses, incorporating uncertainty in dietary and metabolic data and relative risks by age and sex. Robustness of findings was evaluated by sensitivity to varying feasible optimal levels of each risk factor.

**Results:**

In 2010, high systolic blood pressure (SBP) and suboptimal diet were the largest contributors to cardiometabolic deaths in Brazil, responsible for 214,263 deaths (95% uncertainty interval [UI]: 195,073 to 233,936) and 202,949 deaths (95% UI: 194,322 to 211,747), respectively. Among individual dietary factors, low intakes of fruits and whole grains and high intakes of sodium were the largest contributors to cardiometabolic deaths. For premature cardiometabolic deaths (before age 70 years, representing 40% of cardiometabolic deaths), the leading risk factors were suboptimal diet (104,169 deaths; 95% UI: 99,964 to 108,002), high SBP (98,923 deaths; 95%UI: 92,912 to 104,609) and high body-mass index (BMI) (42,643 deaths; 95%UI: 40,161 to 45,111).

**Conclusion:**

suboptimal diet, high SBP, and high BMI are major causes of cardiometabolic death in Brazil, informing priorities for policy initiatives.

## Introduction

Brazil is undergoing rapid economic, demographic and behavioral transitions that are producing major impacts on health. Cardiometabolic diseases including cardiovascular disease (CVD) and type 2 diabetes are the major causes of mortality, leading to nearly 35% of deaths among Brazilian adults [1]. National trends show increasing prevalence of suboptimal levels of well- established cardiometabolic risk factors over last three decades. Trends in national food availability and consumption demonstrate shifts toward unhealthy dietary patterns characterized by higher intakes of processed foods, refined grains, and sugar sweetened beverages; and lower intakes of healthy, minimally processed foods such as fish, fruits, and vegetables [2, 3]. At the same time, the prevalence of adverse metabolic risk factors such as obesity are rising, with a 2–6 fold increase in prevalence of overweight and obesity among adults over the last four decades [4]. Approximately one-third of Brazilian adults have dyslipidemia [5], and about one-third have hypertension [6]. Clearly, the prevalence of suboptimal levels of chronic disease risk factors, particularly those related to poor diet and metabolic health, is substantial, leading to increasing burden on cardiometabolic mortality among Brazilian adults.

To design evidence-informed policies to address this problem, there is a need for clear, consistent data on burdens due to specific risk factors. Yet, the impact of major dietary and metabolic risk factors on cardiometabolic mortality in Brazil according to sex and age has not been clearly established. To address these crucial questions, we used data from the 2010 Global Burden of Diseases, Injuries, and Risk Factors (GBD) Study to evaluate the impact of 11 dietary and 4 metabolic risk factors on cardiometabolic mortality in Brazil in 2010 by sex and age.

## Methods

The 2010 GBD Study is an international collaborative effort since 1990 to produce comprehensive and comparable estimates of the burdens of diseases, injuries and risk factors in 187 countries and territories [7, 8]. Details on methods and standardized data collection protocol have been previously described [9, 10]. We conducted a population-level comparative risk assessment analysis within Brazil for 15 dietary and metabolic risk factors to quantify the overall burdens of disease due to each factor by age and sex.

### Assessment of Risk Factors

A total of 15 major modifiable dietary and metabolic risk factors (11 dietary and 4 metabolic factors) were selected based on probable or convincing evidence for causal effects on coronary heart disease (CHD), stroke or diabetes [11–15]. Mean and standard deviation of dietary exposures by age and sex were obtained from the Brazilian National Dietary Survey (NDS), the dietary component of the 2008–09 Household Budget Survey (POF), which included approximately 55,000 households nationwide. A subset including 25% of the households included in the POF was randomly selected for the dietary survey [16, 17]. In total 34,003 Brazilian adults and children ≥10y of age participated in the NDS, providing two days of dietary records (non-response rate:19%). These data were combined with food disappearance information from the Food and Agriculture Organization database to provide national dietary estimates. Data sources for dietary and metabolic exposures from nationally representative surveys and/or epidemiological studies are shown in Table 1.

**Table 1:** Metabolic and dietary risk factors, their definitions, data sources, optimal levels and cardiometabolic disease outcomes (coronary heart disease, stroke, diabetes and other CVD).

Risk factor (definitions)	Data sources^1^	Optimal level^2^	Disease Outcomes^3^
*Dietary Factors*			
Low intake of fruits	Brazilian Household Budget Survey (2008–2009)	300 ± 30 g/d	IHD, ischemic stroke, hemorrhagic stroke
Low intake of whole grains (food ≥ 1.0 g fiber per 10 g of carbohydrate)		2.5 (50 g) ± 0.25 servings/d	IHD, ischemic stroke, hemorrhagic stroke, diabetes
High intake of sodium^4^		2000 ± 200 mg/d	Blood pressure-mediated effect on IHD, ischemic stroke, hemorrhagic stroke, hypertensive heart disease, aortic aneurism, rheumatic heart disease, inflammatory heart disease, other CVD
Low intake of nuts and seeds		16 ± 1.6 g/day	IHD, diabetes
Low intake of vegetables and beans (excluding vegetable juices, starchy e.g. potatoes, corn, and salted or pickled vegetables); includes intakes of beans and other legumes, except soy milk)		400 ± 40 g/d¥ [18]	IHD, ischemic stroke, hemorrhagic stroke
High intake of processed meats		0 g/d	IHD, diabetes
Low intake of fish and shellfish		50 ± 5 g/day	IHD, ischemic and hemorrhagic stroke
High intake of trans-fats (mainly partially hydrogenated vegetable oils and ruminant products)		0.5 ± 0.05% of total calories[19]	IHD
Low PUFA intake as a replacement for SFA		12 ± 1.2% of total calories^5^[20]	IHD
High intake of red meat (unprocessed) excluding poultry, fish, eggs and all processed meat		100 ± 10 g/week	diabetes
High intake of sugar-sweetened beverages (≥ 50 kcal per 8 oz (226.8 g) serving, excluding 100% fruit and vegetable juices)		0 g/d	Direct effect on DM, BMI-mediated effects on IHD, ischemic stroke, diabetes, hypertensive heart disease
*Metabolic factors*			
High fasting plasma glucose	Ramos et al. 1998; Barreto et el. 2001; Marcopito et al., 2005; Makdisse et al. 2008; Marquezine et al., 2008	5.3 ± 0.3 mmol/L	IHD, ischemic stroke, hemorrhagic stroke, diabetes
High total serum cholesterol	Ramos et al. 1998; Fornes et al., 2002; Marcopito et al., 2005; Makdisse et al. 2008; Marquezine et al., 2008	4.0 ± 0.9 mmol/L	IHD, ischemic stroke
High systolic blood pressure	De Lolio et al., 1990; Ramos et al. 1998; Barreto et el. 2001; Freitas et el. 2001; Moraes et al., 2003; Lessa et al.,2006; Marcopito et al., 2005; Hartmann et al., 2007; Pereira et al. 2007; Castro et at. 2007; Marquezine et al., 2008	115 ± 6 mm Hg	IHD, ischemic stroke, hemorrhagic stroke, hypertensive heart disease, aortic aneurism, rheumatic heart disease, inflammatory heart disease, other CVD
High BMI	Moraes et al. (2003), Barreto et al. (2001), Makdisse et al., 2008	23 ± 1 kg/m^2^ [21, 22]	IHD, ischemic stroke, DM, hypertensive heart disease

IHD: ischemic heart disease, CVD: cardiovascular disease, DM: diabetes mellitus; BMI: body-mass index; PUFA: polyunsaturated fatty acids

^1^ data sources for risk factor exposures from nationally representative surveys and/or epidemiological studies conducted in Brazil.

^2^or dietary risks, a population SD of 10% of the mean was utilized, while for metabolic risks, the population SD of the optimal distribution was determined using a regression evaluating the mean to SD relationship of the corresponding risk factor [23].

^3^ Cardiometabolic diseases with convincing or probable evidence of an etiologic association with risk factors of interest [9].

^4^ High sodium intake was included as a risk factor for aortic aneurism, rheumatic heart disease and inflammatory heart disease based on evidence on the influence of elevated blood pressure on mortality from (not onset or incidence of) these outcomes. In other words, higher BP increases risk of death once these conditions have occurred

^5^ as a replacement for saturated fat.

We evaluated national distributions of body-mass index (BMI), total cholesterol, fasting plasma glucose, and systolic blood pressure (SBP) as previously described [11–14]. Information on exposure levels of metabolic risk factors was obtained from 13 observational studies including Brazilian adults conducted between 1990 and 2008 (Table 1). Age-integrating Bayesian hierarchical modeling methods were used to derive estimates of mean levels of each risk factor and their 95% uncertainty interval (UI) separately by age and sex subgroups. For each exposure, the primary model inputs were nationally representative survey data and exposure levels from epidemiologic studies for each sex and age group, data on the numbers of subjects in each stratum; survey-level indicator covariates for sampling representativeness, food disappearance information from the Food and Agriculture Organization, and country, region (21 regions), and super-region (7 groupings of regions) random effects. This model leverages the interrelationshps between all data to inform final estimates, while appropriately allowing strongest input to Brazil-specific data. [15, 24–26]. Since information on the inherent correlation between age and sex groups resulting from the same underlying Bayesian models was not available, we have not generated p-values for comparison of exposure estimates across subgroups. 95% UIs can be used for informal inference on comparisons between groups: i.e., if the mean value of one estimate is outside the 95% UI of another, these could be considered nominally significantly different.

### Etiological Effects of Risk Factors on Mortality

The relative risks (RR) of CHD, stroke, and diabetes mortality or incidence for each dietary and metabolic risk factor were obtained from published systematic reviews and meta-analyses of randomized controlled trials or prospective cohort studies [27]. When no recent systematic review or meta-analysis were available, de novo meta-analyses were conducted [28]. For these analyses, we used evidence available through November 2014. Most observational studies included had adjusted for relevant potential confounders such as age, sex, race, socioeconomic status, education, physical activity, smoking and alcohol consumption. Etiological effects of metabolic risk factors were estimated using pooled analyses of large cohort projects [29]. For each risk factor-disease pair, we used consistent age-varying distributions of RRs as previously described, incorporating smaller relative effects with increasing age [29]. RRs for both sexes combined was used for men and women, since results from meta-analyses indicated no material differences in RRs by sex [29].

### Optimal Risk Factor Levels

In order to estimate deaths due to suboptimal distributions of risk factors, the optimal distribution was defined as the exposure associated with the lowest possible disease risk that was feasible and observed in some populations, based on current evidence from epidemiologic studies [12, 15, 23, 29] (Table 1). Optimal intake distribution for dietary factors with protective effects (e.g. fruits, vegetables, whole grains) was defined as the intakes to which beneficial effects may plausibly continue based on evidence from current epidemiological studies. For metabolic risk factors and dietary factors with harmful effects, optimal levels were defined as the exposure levels associated with the lowest level of harm seen in observational, and when relevant randomized trials as long as the selected level was also observed at the population level [29]. The standard deviation of the optimal levels was determined using the mean to SD regression co-efficient [8, 15, 23].

### Disease-specific Deaths

The number of deaths due to CHD (ICD-10 codes I20–I25), ischemic stroke (I63, I65–I67, I69.3), hemorrhagic stroke (I60-62, I69.0–2), hypertensive heart disease (I11–I13), aortic aneurysm (I71), rheumatic heart disease (I01, I02.0, I05–I09), inflammatory heart disease (I33, I42), other CVDs, and diabetes (E10–E14) for 2010 were obtained from the GBD 2010 mortality database which provides annual reported data on mortality statistics by country, age, sex, and cause of deaths [30].

### Estimating Mortality Attributable to Risk Factors

The population-attributable fraction PAF was estimated as:

$$PAF=\frac{\int_{x=0}^{m}RR(x)P(x)dx-\int_{x=0}^{m}RR(x)P\prime (x)dx}{\int_{x=0}^{m}RR(x)P(x)dx}$$

where x is the exposure level, P(x) is the actual distribution of exposure in the population, P′(x) is the optimal exposure distribution; RR(x) is the relative risk of mortality at exposure level x, and m is the maximum exposure level. We calculated the number disease-specific deaths attributable to a risk factor by multiplying its PAF by total disease-specific mortality.

We estimated the number of deaths attributed to suboptimal diet by computing the combined population attributable fraction for dietary risk factors based on the following equation: ${PAF}_{subdiet}=1\prod_{i=1}^{11}\left(1-{PAF}_{i}\right)$, where PAFi is the individual dietary risk factor, assuming that the contribution of each component is multiplicative; i.e. that the individual dietary contributions are independent [8]. We conducted all analyses separately by sex and age groups (25–29, 30–34, 35–39, 40–44, 45–49, 50–54, 55–59, 60–64, 65–69, 70–74, 75–79, and 80+ years).

Due to joint distributions, multicausality, interaction, and because the effects of some risk factors are partly mediated through other risk factors, the number of deaths attributable to different risk factors cannot be summed. For example, the burden due to high SBP is partly mediated by high sodium intake and low intakes of fruits and vegetables; whereas much of the burden due to high BMI is mediated by high SBP, cholesterol, and glucose. Thus, the numbers of deaths attributable to each risk factor should be considered the total numbers of deaths due to this factor, including its upstream determinants and downstream mediators.

### Uncertainty and Sensitivity Analyses

We quantified statistical uncertainty using probabilistic simulation analyses. Using the Monte Carlo simulations, we drew 1000 observations (unbiased random samples) from the normal distribution of exposure, optimal intake levels, and mortality, as well as a log-normal distribution of disease-specific RRs. We reported 95% UI for deaths related to a risk factor based on the 2.5^th^ and 97.5^th^ percentile of the resulting distributions of attributable deaths. These UI include all sources of uncertainty from each component of the analysis, including sampling error and uncertainties associated with model parameters [31]. In sensitivity analyses, we evaluated different optimal levels for each metabolic risk factor. All analyses were conducted using R version 2.15.0.

## Results

### Dietary consumption

In 2010, the mean consumption of all evaluated healthful foods by Brazilian adults was below optimal levels, while intakes of unhealthful dietary factors were higher than optimal (Table 2). The largest differences between mean intakes and optimal levels were observed for fruits, whole grains, and nuts, with mean national intakes only between 8% to 30% of optimal. Among unhealthy dietary factors, mean trans-fat intake exceeded the optimal level by 200%, while sodium consumption was over 100% greater than the WHO recommendation of 2.0g/day. In contrast, intakes of fish and vegetables/beans were each closer to optimal levels. Compared to middle-aged and older adults (45y+), younger adults showed lower consumption of healthful foods such as fruits, vegetables and fish, and higher consumption of sugar-sweetened beverages, red and processed meats. Similarly, women exhibited generally healthier dietary habits than men (Table 2).

**Table 2:** Age-adjusted national means^1^ (95% uncertainty intervals) of dietary and metabolic risk factors among Brazilians, 2010.

	Optimal level	Men	Women	25-44y	45-69y	70+y
*Dietary Factors (g/day)*						
**Fruits**	300	89.5 (85.2, 93.6)	107.5 (103.3, 112)	92.4 (87.9, 96.7)	105.1 (100.4, 110)	113.4 (108.1, 118.7)
**Vegetables and beans**	400	260 (251, 269)	264 (256, 272)	252 (244, 261)	276 (265, 284)	269 (259, 280)
**Whole grains**	125	13.7 (13.1, 14.4)	14.5 (13.9, 15.2)	12.5 (11.9, 13.2)	15.4 (14.8, 16)	19.2 (18.3, 20.1)
**Nuts**	16	2.1 (2, 2.2)	2.3 (2.2, 2.4)	2.1 (2, 110)	2.4 (2.3, 2.5)	1.9 (1.8, 2)
**Fish**	50	41.3 (39.9, 42.9)	40.9 (39.5, 42.3)	37.2 (35.8, 38.5)	45.7 (44.1, 47.4)	41.3 (39.9, 0)
**Red meat**	14	96 (93, 99)	87 (84, 89)	92 (89, 95)	80 (78, 83)	85 (82, 88)
**Processed meat**	0	27.8 (26.5, 29)	23.7 (22.6, 24.8)	27.5 (26.2, 28.8)	24.1 (23, 25.2)	20.7 (19.7, 21.7)
**Sugar sweetened beverages**	0	121 (115, 128)	107 (102, 113)	146 (139, 153)	79 (75, 84)	58 (55, 61)
**PUFA** (%kcal)	12	6.9 (1.6, 4382.3)	7 (6.9, 7.2)	7 (6.9, 7.1)	7 (6.8, 7.1)	6.6 (6.6, 6.8)
**SFA** (%kcal)	—	8.8 (8.7, 9)	9 (8.8, 9.2)	9 (8.8, 9.1)	8.9 (8.7, 9)	8.9 (8.7, 9.1)
**trans-fat (%kcal)**	0.5	1.8 (1.7, 1.8)	1.6 (1.5, 1.7)	1.8 (1.8, 1.9)	1.8 (1.7, 1.9)	1.7 (1.6, 1.8)
**Sodium** (mg/day)	2,000	4323 (4243, 4404)	3934 (3864, 4002)	4094 (4013, 4174)	4154 (4079, 4236)	4144 (4062, 4226)
*Metabolic Risk Factors*						
**Body-mass inde**x (kg/m^2^)	23	26.2 (26, 26.5)	26.5 (26.2, 26.7)	25.7 (25.5, 26)	27.2 (26.9, 27.5)	26.5 (26.2, 26.9)
**Systolic blood pressure** (mmHg)	115	133.5 (131.6, 135.1)	124.1 (122.4, 125.8)	121.0 (119.3, 122.7)	135.8 (133.6, 137.9)	146.1 (142.3, 149.6)
**Fasting plasma glucose** (mmol/l)	5.3	5.6 (5.3, 5.8)	5.5 (5.3, 5.7)	5.2 (5, 5.5)	5.9 (5.6, 6.2)	5.9 (5.6, 6.3)
**High total cholesterol** (mmol/l)	4	4.8 (4.6, 5)	5.6 (5, 6.2)	4.5 (4.3, 4.7)	5.2 (4.9, 5.5)	5.4 (5, 5.8)

^1^Means are age-adjusted to the 2010 Brazilian population distribution.

### Metabolic risk factors

National mean levels of metabolic risk factors exceeded optimal levels, with largest differences for SBP (mean [95%UI]: 133.5 mmHg [131.6–135.1] in men and 124.1 mmHg [122.4–125.8] in women; optimal level: 115 mmHg) and BMI (mean [95%UI]: 26.2kg/m^2^ [26.0, 26.5] in men and 26.5kg/m^2^ [26.2, 26.7] in women; optimal: 23kg/m^2^;) (Table 2). When comparing age-adjusted estimates, Brazilian men had higher mean SBP levels and lower mean total cholesterol levels than women (Table 2). On the other hand, mean BMI and fasting plasma glucose concentrations were similar in men vs. women. Mean levels of each of these metabolic risk factors increased with age.

### Cardiovascular and Metabolic Death

In 2010, 446,084 total cardiometabolic deaths occurred in Brazil, with 161,261 (36.1%) due to ischemic heart disease, 144,295 (32.3%) to stroke (ischemic and hemorrhagic), and 53,353 (12%) to diabetes. About 4 in 10 (38%) of these deaths occurred prematurely, i.e. before the age of 70y. The leading cause of premature cardiometabolic deaths among Brazilians was ischemic heart disease (67%), followed by hemorrhagic stroke (19%) and diabetes (13%). Major causes of cardiometabolic deaths were similar among men and women (Table 3).

**Table 3:** Deaths among Brazilians 25+y in 2010 by disease outcome.

Disease Outcome	Alln	Men^λ^	Women	25-44y	45-69y	70+y
	n	n (%)	n (%)	n (%)	n (%)	n (%)
**Ischemic Heart Disease**	161,261	87,603 (54)	73,658 (46)	6,367 (4)	58,441 (36)	96,453 (60)
**Ischemic Stroke**	73,216	35,994 (49)	37,223 (51)	886 (1)	14,044 (19)	58,286 (80)
**Hemorrhagic Stroke**	67,079	33,972 (51)	33,107 (49)	4,311 (6)	28,183 (42)	34,585 (52)
**Diabetes Mellitus**	53,353	22,825 (43)	30,528 (57)	1,740 (3)	19,365 (36)	32,248 (60)
**Hypertensive Heart Disease**	34,033	15,861 (47)	18,171 (53)	1,012 (3)	10,029 (29)	22,992 (68)
**Aortic Aneurism**	7,009	4,385 (63)	2,623 (37)	338 (5)	2,894 (41)	3,777 (54)
**Inflammatory Heart Disease**	23,336	13,363 (57)	9,973 (43)	1,886 (8)	8,480 (36)	12,970 (56)
**Rheumatic Heart Disease**	5,506	2,070 (38)	3,437 (62)	650 (12)	1,661 (30)	3,196 (58)
**Other CVD**	21,291	9,649 (45)	11,642 (55)	1,305 (6)	6,220 (29)	13,765 (65)

Source: Institute for Health Metrics and Evaluation.

^λ^ stratum-specific number of deaths and % for each disease outcome.

### The impact on suboptimal risk factors on cardiometabolic mortality

**Fig 1:**
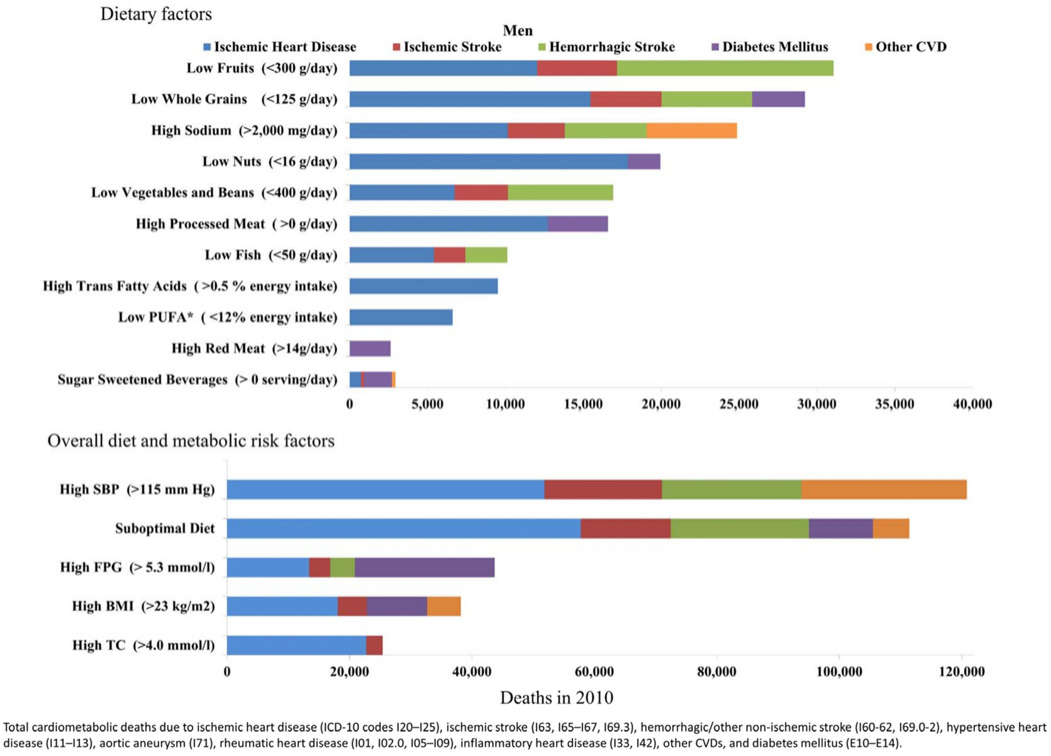
Cardiometabolic deaths attributable to suboptimal dietary and metabolic factors in Brazilian men in 2010.

**Fig 2:**
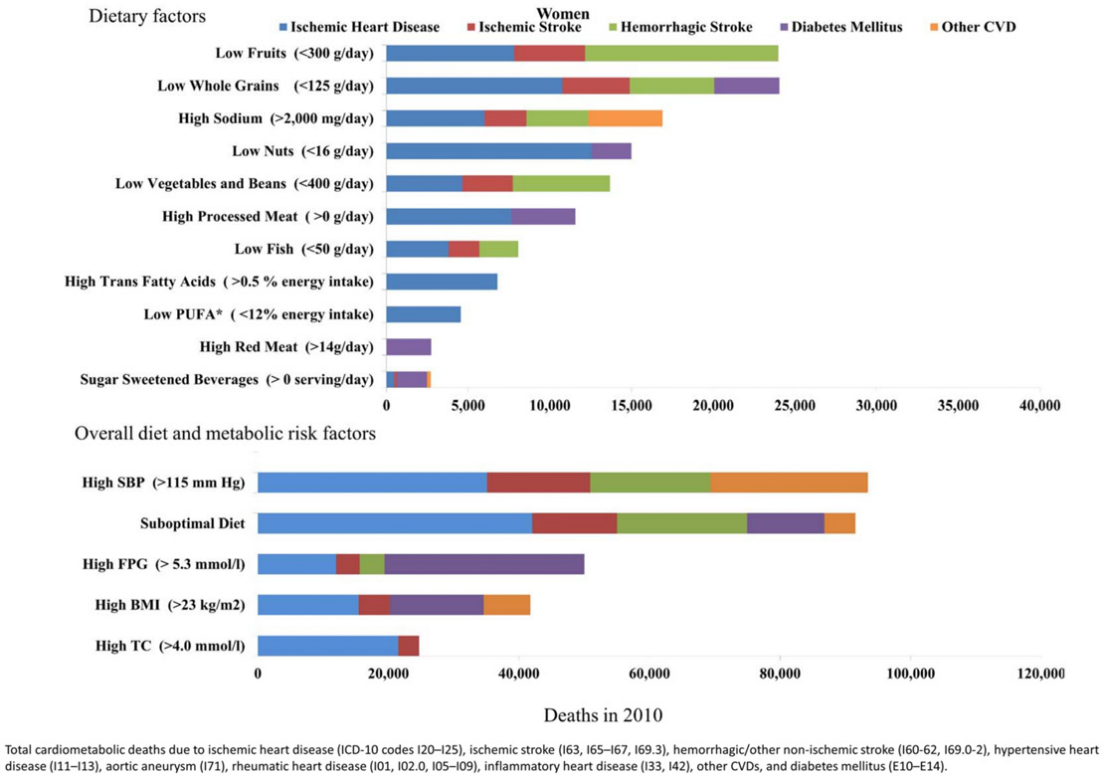
Cardiometabolic deaths attributable to suboptimal dietary and metabolic factors in Brazilian women in 2010.

The impact of suboptimal diet on cardiometabolic mortality in Brazil in 2010 was substantial, estimated to account for nearly 45% of cardiometabolic deaths (202,949 deaths; 95% UI, 194,322 to 211,747). Among individual dietary factors, low intake of fruits was the largest contributor to cardiometabolic mortality, leading to 55,051 deaths (95% UI, 50,534 to 59,658). Low intake of whole grains (53,269 deaths; 95% UI, 50,594 to 56,072) and high intake of sodium (41,751 deaths; 95% UI, 26,190 to 56,070) were the second and third leading dietary factors contributing to cardiometabolic death in the country, followed by low intake of nuts (34,923 deaths; 95%UI, 31,532 to 38,446), low intake of vegetables and beans (30,613 deaths; 95%UI, 27,412 to 33,727)] and high intake of processed meats (30,613 deaths; 95%UI, 27,412 to 33,727)] (Table 4). The number of cardiometabolic deaths (per million adults) was consistently higher in men compared to women, particularly for sodium (463 deaths per million adults; 95%UI, 293 to 626 in men; 290 deaths per million adults; 95%UI, 185 to 401 in women) (Table 4, Figs 1 and 2).

**Table 4:** Cardiometabolic deaths^1^ (95% uncertainty intervals) in Brazil in 2010 attributable to dietary and metabolic risk factors.

	Total	Men	Women	25-44y	45-69y	70+y
Total cardiometabolic (CMD) deaths^2^	446,113	225,729	220,384	18,496	149,308	278,308
Dietary Risk Factors						
**Suboptimal Diet**^3^						
CMD deaths	202949	111434	91515	11995	92175	98780
	(194322, 211747)	(106164, 116471)	(86723, 96391)	(11459, 12519)	(88177, 95688)	(93168, 104326)
Deaths per million	1815 (1738, 1894)	2076 (1978, 2169)	1575 (1492, 1659)	198 (189, 206)	2187 (2092, 2270)	10992 (10368, 11609)
**Low intake of fruits** (< 300g/day)						
CMD deaths	55051	31073	23978	4559	26645	23848
	(50534, 59658)	(27786, 34330)	(21239, 27007)	(3860, 5294)	(23551, 29425)	(20652, 27224)
Deaths per million^4^	492 (452, 534)	579 (518, 639)	413 (366, 465)	75 (64, 87)	632 (559, 698)	2654 (2298, 3029)
% total CMD deaths^5^	12	14	11	25	18	9
**Low intake of whole grains** (< 125 g/day)						
CMD deaths	53269	29223	24046	3989	25535	23745
	(50594, 56072)	(27193, 31342)	(22236, 25914)	(3530, 4454)	(23681, 27485)	(21530, 25875)
Deaths per million^4^	476 (453, 502)	544 (507, 584)	414 (383, 446)	66 (58, 73)	606 (562, 652)	2642 (2396, 2879)
% total CMD deaths^5^	12	13	11	22	17	9
**High intake of sodium** (>2000mg/day)^6^						
CMD deaths	41751	24870	16880	2127	22875	16749
	(26190, 56070)	(15715, 33590)	(10741, 23312)	(873, 3415)	(15100, 29842)	(9995, 23527)
Deaths per million^4^	373 (234, 502)	463 (293, 626)	290 (185, 401)	35 (14, 56)	543 (358, 708)	1864 (1112, 2618)
% total CMD deaths^5^	9.4	11.0	7.7	11.5	15.3	6.0
**Low intake of nuts** (< 16 g/day)						
CMD deaths	34923	19938	14986	2512	17270	15141
	(31532, 38446)	(17417, 22637)	(12646, 17178)	(2057, 3014)	(14986, 19617)	(12615, 17672)
Deaths per million^4^	312 (282, 344)	371 (324, 422)	258 (218, 296)	41 (34, 50)	410 (356, 465)	1685 (1404, 1967)
% total CMD deaths^5^	8	9	7	14	12	5
**Low intake of vegetable and beans** (<400g/day)						
CMD deaths	30613	16927	13686	2655	14199	13759
	(27412, 33727)	(14529, 19405)	(11466, 16052)	(2093, 3207)	(12110, 16260)	(11537, 16335)
Deaths per million^4^	274 (245, 302)	315 (271, 361)	236 (197, 276)	44 (35, 53)	337 (287, 386)	1531 (1284, 1818)
% total CMD deaths^5^	7	7	6	14	10	5
**High intake of processed meat** (>0 g/day)						
CMD deaths	28145	16591	11554	2471	14510	11164
	(24027, 32808)	(13262, 20304)	(9118, 14142)	(1787, 3193)	(11531, 17538)	(8471, 14030)
Deaths per million^4^	252 (215, 293)	309 (247, 378)	199 (157, 243)	41 (29, 53)	344 (274, 416)	1242 (943, 1561)
% total CMD deaths^5^	6	7	5	13	10	4
**Low intake of fish** (< 50g/day)						
CMD deaths	18188	10114	8074	1576	8622	7990
	(15887, 20320)	(8249, 11900)	(6747, 9479)	(1227, 1976)	(7193, 10150)	(6355, 9555)
Deaths per million^4^	163 (142, 182)	188 (154, 222)	139 (116, 163)	26 (20, 33)	205 (171, 241)	889 (707, 1063)
% total CMD deaths^5^	4	4	4	9	6	3
**High intake of trans-fat** (>0.5kcal/day)						
CMD deaths	16309	9510	6799	1338	8368)	6603
	(14898, 17787)	(8381, 10603)	(5861, 7729)	(1100, 1606)	(7395, 9378	(5579, 7675)
Deaths per million^4^	146 (133, 159)	177 (156, 197)	117 (101, 133)	22 (18, 26)	199 (175, 222)	735 (621, 854)
% total CMD deaths^5^	4	4	3	7	6	2
**Low intake of PUFA** (<12%E)						
CMD deaths	11167	6622	4544	827	5535	4804
	(9654, 12762)	(5387, 7741)	(3600, 5580)	(618, 1052)	(4449, 6577)	(3720, 5975)
Deaths per million^4^	100 (86, 114)	123 (100, 144)	78 (62, 96)	14 (10, 17)	131 (106, 156)	535 (414, 665)
% total CMD deaths^5^	3	3	2	4	4	2
**High intake of red meat** (>14 g/day)						
CMD deaths	5363	2633	2730	363	2748	2251
	(4322, 6445)	(1889, 3407)	(1917, 3576)	(234, 508)	(1993, 3585)	(1451, 3092)
Deaths per million^4^	48 (39, 58)	49 (35, 63)	47 (33, 62)	6 (4, 8)	65 (47, 85)	251 (162, 344)
% total CMD deaths^5^	1	1	1	2	2	1
**High intake of sugar sweetened beverages** (>0 g/day)						
CMD deaths	5666	2951	2715	578	2849	2239
	(4471, 6829)	(2268, 3615)	(2134, 3289)	(455, 712)	(2201, 3494)	(1709, 2780)
Deaths per million^4^	51 (40, 61)	55 (42, 67)	47 (37, 57)	10 (8, 12)	68 (52, 83)	249 (190, 309)
% total CMD deaths^5^	1.3	1.3	1.2	3.1	1.9	0.8
Metabolic Risk Factors						
**High systolic blood pressure** (>115 mmHg)						
CMD deaths	214263	120833	93430	8565	90359	115340
	(195073, 233936)	(108621, 133678)	(80120, 108208)	(7289, 9794)	(84495, 95881)	(97030, 133417)
Deaths per million^4^	1917 (1745, 2093)	2251 (2023, 2490)	1608 (1379, 1862)	141 (120, 161)	2144 (2004, 2275)	12835 (10798, 14847)
% total CMD deaths^5^	48.0	53.5	42.4	46.3	60.5	41.4
**High fasting plasma glucose** (> 5.3mmol/L)						
CMD deaths	93693	43686	50007	2919	35978	54796
	(85562, 102756)	(38104, 49766)	(44191, 56848)	(2315, 3622)	(32088, 40127)	(47530, 63208)
Deaths per million^4^	838 (765, 919)	814 (710, 927)	861 (760, 978)	48 (38, 60)	853 (761, 952)	6098 (5289, 7034)
% total CMD deaths^5^	21.0	19.4	22.7	15.8	24.1	19.7
**High body-mass inde**x (>23 kg/m2)						
CMD deaths	79914	38165	41749	4072	38571	37272
	(74065, 86444)	(34781, 41576)	(36829, 46907)	(3570, 4603)	(36097, 41022)	(32034, 43078)
Deaths per million^4^	715 (663, 773)	711 (648, 774)	718 (634, 807)	67 (59, 76)	915 (856, 973)	4148 (3565, 4794)
% total CMD deaths^5^	17.9	16.9	18.9	22.0	25.8	13.4
**High total cholesterol** (> 4.0 mmol/L)						
CMD deaths	50129	25411	24718	2615	23905	23610
	(36020, 69732)	(17650, 35630)	(14037, 41930)	(1745, 3555)	(18417, 29355)	(10917, 42825)
Deaths per million^4^	448 (322, 624)	473 (329, 664)	425 (242, 722)	43 (29, 59)	567 (437, 696)	2627 (1215, 4766)
% total CMD deaths^5^	11.2	11.3	11.2	14.1	16.0	8.5

^1^Due to joint distributions, multicausality, interaction, and because the effects of some risk factors are partly mediated through other risk factors, the number of deaths attributable to different risk factors cannot be summed. For example, part of the burden due to high BP is due to high sodium intake and low intakes of fruits and vegetables; whereas much of the burden due to high BMI is mediated by high BP, cholesterol, and glucose. Thus, the numbers of deaths attributable to each risk factor should be considered the total numbers of deaths due to this factor, including its upstream determinants and downstream mediators.

^2^Total cardiometabolic deaths due to CHD (ICD-10 codes I20–I25), ischemic stroke (I63, I65–I67, I69.3), hemorrhagic/other non-ischemic stroke (I60-62, I69.0–2), hypertensive heart disease (I11–I13), aortic aneurysm (I71), rheumatic heart disease (I01, I02.0, I05–I09), inflammatory heart disease (I33, I42), other CVDs, and diabetes mellitus (E10–E14).

^3.^ Burden from suboptimal diet was calculated by computing the combined population attributable fraction for dietary risk factors assuming that the contribution of each component is multiplicative.

^4^ Based on the number of people in the same stratum of the population (e.g. men, 25-44y etc).

^5^ Based on the total number of deaths in the same stratum of the population (e.g. men, 25-44y etc).

^6^only mediated effects through blood pressure.

When evaluating the impact of metabolic risk factors on cardiometabolic mortality, we found that high SBP was the largest contributor to cardiometabolic mortality in 2010, leading to 214,263 deaths (95%UI, 195,073 to 233,936) in the period. High fasting plasma glucose was the second largest metabolic risk factor contributing to cardiometabolic death (93,693 cardiometabolic deaths; 95%UI 85,562 to 102,756), followed by high BMI (79,914 deaths; 95%UI, 74,065 to 86,444) and high total cholesterol (50,129 deaths; 95%UI, 36,020 to 69,732). Overall, cardiometabolic mortality rates attributable to metabolic risk factors were similar in males and females, except for high SBP, where the number of cardiometabolic deaths (per million adults) in men was substantially higher than those observed in women (2,251 deaths per million adults; 95%UI, 2,023 to 2,490 in men; 1,608 deaths per million adults; 95%UI, 1,379 to 1,862 in women).

### The impact of suboptimal risk factors on premature cardiometabolic mortality

**Fig 3:**
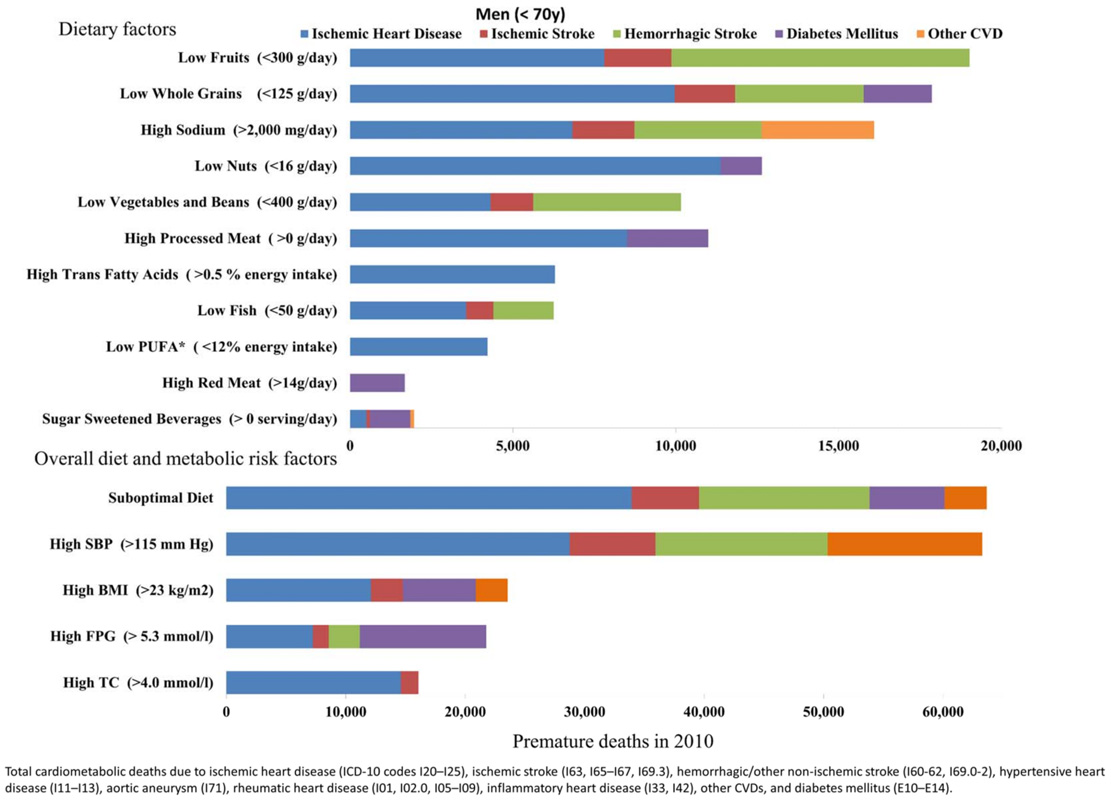
Premature cardiometabolic deaths attributable to suboptimal dietary and metabolic factors in Brazilian men 25-69y in 2010.

**Fig 4:**
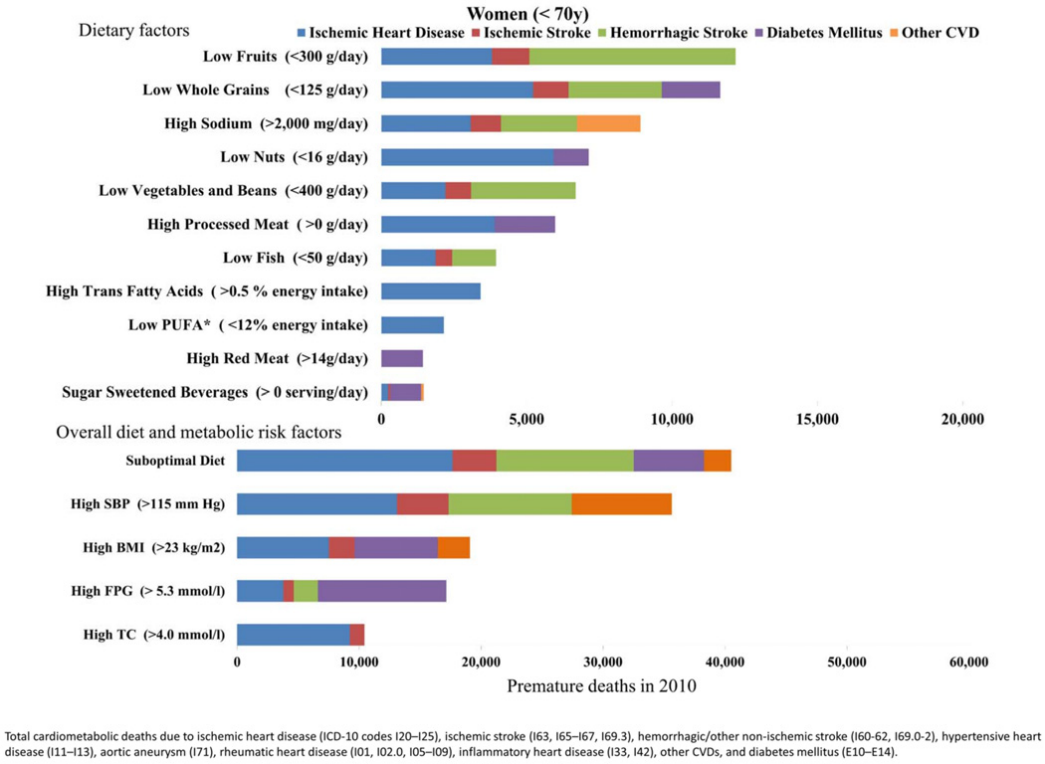
Premature cardiometabolic deaths attributable to suboptimal dietary and metabolic factors in Brazilian women 25-69y in 2010.

Suboptimal diet was the largest contributor to premature cardiometabolic death in Brazil, accounting for 62% of premature deaths (104,169 premature cardiometabolic deaths,95%UI, 99,964 to 108,002) among men and women in 2010 (Table 5, Figs 3 and 4). The second leading risk factor for early death was high SBP, followed by high BMI, which was the fourth leading risk factor for mortality in all age groups. The relative greater impact of high BMI on premature mortality was mostly due to suboptimal BMI among adults between 45 and 69 years of age.

**Table 5:** Premature cardiometabolic deaths^1^ (95% uncertainty intervals) attributable to suboptimal dietary and metabolic risk factors in Brazil in 2010 among adults 25-69y

	Total	Men	Women
Total cardiometabolic (CMD) deaths^2^	167,804	99,821	67,983
Dietary Risk Factors			
**Suboptimal diet**^3^			
CMD deaths	104169 (99964, 108002)	63648 (60573, 66229)	40521 (38813, 42260)
Deaths per million^4^	1013 (972, 1051)	1277 (1215, 1328)	765 (733, 798)
% total CMD deaths^5^	62	64	60
**Low intake of fruits** (< 300g/day)			
CMD deaths	31203 (28049, 34167)	19021 (16570, 21445)	12183 (10447, 13905)
Deaths per million^4^	304 (273, 332)	382 (332, 430)	230 (197, 263)
% total CMD deaths^5^	19	19	18
**Low intake of whole grains** (< 125 g/day)			
CMD deaths	29524 (27597, 31429)	17864 (16131, 19538)	11660 (10671, 12609)
Deaths per million^4^	287 (268, 306)	358 (324, 392)	220 (202, 238)
% total CMD deaths^5^	18	18	17
**High intake of sodium**^6^ (>2000mg/day)			
CMD deaths	25002 (16514, 32818)	16093 (10420, 21081)	8909 (5838, 11804)
Deaths per million^4^	243 (161, 319)	323 (209, 423)	168 (110, 223)
% total CMD deaths^5^	14.9	16.1	13.1
**Low intake of nuts** (< 16 g/day)			
CMD deaths	19782 (17533, 22195)	12650 (10639, 14642)	7132 (6009, 8265)
Deaths per million^4^	192 (171, 216)	254 (213, 294)	135 (113, 156)
% total CMD deaths^5^	12	13	10
**Low intake of vegetable and beans** (<400g/day)			
CMD deaths	16854 (14699, 19017)	10166 (8419, 12044)	6688 (5466, 8012)
Deaths per million	164 (143, 185)	204 (169, 242)	126 (103, 151)
% total CMD deaths	10	10	10
**High intake of processed meat** (>0 g/day)			
CMD deaths	16981 (13887, 20103)	11000 (8203, 13894)	5982 (4582, 7495)
Deaths per million^4^	165 (135, 196)	221 (165, 279)	113 (87, 142)
% total CMD deaths^5^	10	11	9
**Low intake of fish** (< 50g/day)			
CMD deaths	10198 (8614, 11776)	6251 (4954, 7672)	3947 (3216, 4754)
Deaths per million^4^	99 (84, 115)	125 (99, 154)	75 (61, 90)
% total CMD deaths^5^	6	6	6
**High intake of trans-fat** (>0.5kcal/day)			
CMD deaths	9706 (8705, 10716)	6288 (5338, 7227)	3418 (2911, 3970)
Deaths per million^4^	94 (85, 104)	126 (107, 145)	65 (55, 75)
% total CMD deaths^5^	6	6	5
**Low intake of PUFA** (<12%E)			
CMD deaths	6362 (5263, 7408)	4220 (3279, 5148)	2143 (1678, 2610)
Deaths per million^4^	62 (51, 72)	85 (66, 103)	40 (32, 49)
% total CMD deaths^5^	4	4	3
**High intake of sugar sweetened beverages** (>0 g/day)			
CMD deaths	3427 (2698, 4125)	1966 (1519, 2425)	1461 (1162, 1786)
Deaths per million^4^	33 (26, 40)	39 (30, 49)	28 (22, 34)
% total CMD deaths^5^	2.0	2.0	2.1
			
**High intake of red meat**(>14 g/day)			
CMD deaths	3111 (2337, 3938)	1679 (1095, 2257)	1433 (931, 1996)
Deaths per million^4^	30 (23, 38)	34 (22, 45)	27 (18, 38)
% total CMD deaths^5^	2	2	2
Metabolic Risk Factors			
**High systolic blood pressure** (>115 mmHg)			
CMD deaths	98923 (92912, 104609)	63289 (58344, 67817)	35634 (32044, 38815)
Deaths per million^4^	962 (904, 1018)	1269 (1170, 1360)	673 (605, 733)
% total CMD deaths^5^	59.0	63.4	52.4
**High body-mass inde**x (>23 kg/m^2^)			
CMD deaths	42643 (40161, 45111)	23548 (21638, 25452)	19094 (17457, 20679)
Deaths per million^4^	415 (391, 439)	472 (434, 511)	361 (330, 391)
% total CMD deaths^5^	25.4	23.6	28.1
**High fasting plasma glucose** (> 5.3mmol/L)			
CMD deaths	38897 (34958, 43046)	21747 (18218, 25158)	17150 (14863, 19571)
Deaths per million^4^	378 (340, 419)	436 (365, 505)	324 (281, 370)
% total CMD deaths^5^	23.2	21.8	25.2
**High total cholesterol** (> 4.0 mmol/L)			
CMD deaths	26520 (21052, 32031)	16081 (11651, 20522)	10438 (7627, 13326)
Deaths per million^4^	258 (205, 312)	323 (234, 412)	197 (144, 252)
% total CMD deaths^5^	15.8	16.1	15.4

^1^Due to joint distributions, multicausality, interaction, and because the effects of some risk factors are partly mediated through other risk factors, the number of deaths attributable to different risk factors cannot be summed. For example, part of the burden due to high BP is due to high sodium intake and low intakes of fruits and vegetables; whereas much of the burden due to high BMI is mediated by high BP, cholesterol, and glucose. Thus, the numbers of deaths attributable to each risk factor should be considered the total numbers of deaths due to this factor, including its upstream determinants and downstream mediators.

^2^Total cardiometabolic deaths due to CHD (ICD-10 codes I20–I25), ischemic stroke (I63, I65–I67, I69.3), hemorrhagic/other non-ischemic stroke (I60-62, I69.0–2), hypertensive heart disease (I11–I13), aortic aneurysm (I71), rheumatic heart disease (I01, I02.0, I05–I09), inflammatory heart disease (I33, I42), other CVDs, and diabetes mellitus (E10–E14).

^3.^ Burden from suboptimal diet was calculated by computing the combined population attributable fraction for dietary risk factors assuming that the contribution of each component is multiplicative.

^4^ Based on the number of people in the same stratum of the population (e.g. men, 25-44y etc)

^5^ Based on the total number of deaths in the same stratum of the population (e.g. men, 25-44y etc).

^6^only mediated effects through blood pressure.

### Sensitivity Analysis

In sensitivity analysis, increasing the optimal level of high SBP from 115 to 120 mmHg decreased the number of SBP-attributable cardiometabolic deaths by about 9% (S1 Table). Lowering the optimal levels of BMI (from 23 to 21 kg/m^2^), fasting plasma glucose (from 5.3 to 4.9 mmol/l), and total cholesterol (from 4.1 to 3.9 mmol/l) increased their respective attributable mortality by 20%-30%.

## Discussion

We have used up-to-date data, consistent and comparable methodology to estimate the impact of 11 dietary and 4 metabolic risk factors on cardiometabolic mortality in Brazil in 2010. Suboptimal diet and high SBP were the largest contributors to cardiometabolic death in Brazil. Among individual dietary factors, low intakes of fruits and whole grains and high intakes of sodium were the major risk factors for cardiometabolic death.

Our dietary consumption estimates are consistent with recent studies reporting lower household availability and mean intakes of fruits, rice, beans, nuts, and fish, and relatively high consumption of processed meat, refined sugar, sugar sweetened beverages, and frozen meals in Brazil [2, 32, 33]. Previous studies reported a more than doubling in consumption of highly processed foods such as sausage, frozen meals and soft drinks in the past 3 decades, while energy from traditional foods such as rice and beans decreased between 10–20% in the same period [3]. Overall, our findings show the gap between current and optimal consumption was highest among younger adults (<45y). If these poor habits continue as these younger Brazilians age, the burden of cardiometabolic deaths due to suboptimal diet is likely to increase dramatically over time.

Although reductions in smoking and improved health care access have contributed to a nearly 30% reduction in CVD mortality between 1996 and 2007 in Brazil [5], our results indicate that the population burdens of major metabolic risk factors remain high, especially as it relates to premature mortality. About 60% of premature cardiometabolic deaths were attributable to high SBP, while 23% were due to high fasting plasma glucose, which suggests the need for appropriate measures to reduce the prevalence of these important metabolic risk factors in the country. Recent reports from the Longitudinal Study of Adult Health, a cohort including ~15,000 Brazilian civil servants aged 35–74 years, found that about 50% of participants with diabetes had not been previously diagnosed or received treatment [34]; nearly 1 of 5 participants with measured hypertension had not been previously diagnosed; and about 47% of participants with measured hypertension had not received treatment for this condition [35]. In our investigation, the large burdens of BP highlight the relevance of excess sodium consumption among Brazilian adults and the potential population benefits of sodium reduction programs. Better BP screening and control with medications, and lifestyle treatment of those with prevalent diabetes and pre-diabetes, are also strongly indicated.

The overall adverse metabolic profile may be explained in part by the high prevalence of overweight and obesity, which was the third leading factor for premature cardiometabolic mortality in the country. Currently nearly 50% of Brazilian adults are overweight and 15% are obese [16, 36]. Importantly, obesity rates in the past four decades have been greater among individuals in lower income groups [37]. Excess adiposity increases blood pressure, dyslipidemia, as well as risk of diabetes, IHD, and stroke [38]. It has been well established that substantial reduction in cardiometabolic risk through adherence to a healthy lifestyle including healthy diet and regular exercise. Our findings emphasize the need to improve diet through increasing consumption of healthy foods including fruits, whole grains nuts and beans, and lowering intakes of unhealthy food components such sodium and processed meats, which will help reduce BMI and blood pressure levels in Brazil.

Brazil has had success at implementing large-scale measures that have improved public health. For example, national immunization programs for yellow fever, small pox and poliomyelitis have nearly eradicated these conditions [39, 40]. In their place, cardiometabolic diseases including CHD, stroke, and diabetes are now the leading causes of mortality and the greatest public health challenges. Launched by the Brazilian Ministry of Health in 2011, the Strategic Action Plan to Tackle non-Communicable Chronic Diseases set 12 national goals for 2022, including a 2% yearly reduction in premature mortality due to non-Communicable Chronic Diseases, as well as improvements to national levels of three dietary targets (fruits, leafy vegetables, sodium) and one metabolic risk factor (BMI), in addition to increasing leisure-time physical activity [41]. Our data suggest that substantial reduction in premature mortality could be achieved by expanding the national goals to include other dietary factors such as whole grains, nuts, beans, and fish, as well as lowering processed meats and trans-fats. Our findings also emphasize the need for additional concerted efforts to improve risk factors among young adults and males, especially.

Our study has several strengths. We used consistent and comparable methods to estimate the impact of key dietary and metabolic risk factors on cardiometabolic mortality in Brazil by age and sex, informing national priorities for prevention strategies. We used up-to-date evidence on effect sizes of diet-disease associations based on meta-analyses. We evaluated sensitivity of our findings to alternative optimal levels of dietary and metabolic risk factors. We accounted for potential age- and sex-differences in the current levels of risk factors, the relative magnitude of their effects cardiometabolic mortality, as well as cause-specific mortality.

Potential limitations should be considered. Although we used RRs for associations of dietary and metabolic risk factors from meta-analyses of observational studies adjusting for relevant confounders, residual confounding may be present. To evaluate the magnitude of potential bias on measures of associations, particularly due to residual confounding by dietary factors, a sensitivity analysis was previously performed comparing GBD estimates to RRs from randomized controlled feeding trials evaluating effects of overall dietary patterns on CVD risk factors (DASH, DASH-sodium, OmniHeart) [27]. There was no substantial differences between effect size estimates used in this analysis compared to those of feeding trials, which indicates that our RRs were not substantially affected by correlation of dietary factors [27]. We used the joint population attributable fraction to estimate the number of cardiometabolic deaths attributable to the combined effect of dietary factors and assumed the intakes of individual dietary factors were independent within each age-sex stratum. This could overestimate the cardiometabolic burden of suboptimal diet. On the other hand, the cardiometabolic burden due to suboptimal diet might be underestimated due to measurement error and over-adjustment in the source studies for potential mediators (e.g., blood pressure, BMI, blood lipids). Finally, we focused on cardiometabolic mortality, and dietary and metabolic risk factors could increase the risk of other diseases, such as cancer. Thus, our estimates are a conservative estimate of the overall burden of disease due to these risk factors in Brazil.

In conclusion, our estimates of the impact of suboptimal dietary and metabolic risk factors on cardiometabolic mortality in Brazil in 2010 indicate that the major risk factors were suboptimal diet, particularly low fruits and whole grains and high sodium, high SBP, high fasting plasma glucose and high BMI. Our findings inform priorities for policy measures to reduce CVD and diabetes mortality in Brazilian adults.

## Supporting Information

S1 Table Cardiometabolic deaths (95% UI) attributable to suboptimal risk factors in Brazil in 2010, estimated using lower reference levels. (DOCX)
