# Supplementary material for: The Impact of Dietary and Metabolic Risk Factors on Cardiovascular Diseases and Type 2 Diabetes Mortality in Brazil
Source: PLoS One. 2016 Mar 18;11(3):e0151503. doi: 10.1371/journal.pone.0151503 (PMC4798497; doi:10.1371/journal.pone.0151503)
Supplement: S1 Table — (DOCX) [file pone.0151503.s001.docx]

| S1 Table: Cardiometabolic deaths^1^ (95% uncertainty intervals) attributable to suboptimal risk factors in Brazil in 2010, estimated using lower reference levels | | | |
| --- | --- | --- | --- |
| Cardiometabolic (CMD) deaths^2^ | Total | Men | Women |
| **High systolic blood pressure** (>120 mmHg) |  |  |  |
| CMD deaths | 195394 (177111, 214507) | 111337 (99607, 124146) | 84056 (71727, 98176) |
| Deaths per million^3^ | 1748 (1584, 1919) | 2074 (1855, 2312) | 1447 (1234, 1689) |
| % total CMD deaths^4^ | 43.8 | 49.3 | 38.1 |
| **High body-mass inde**x (>21 kg/m2) |  |  |  |
| CMD deaths | 102223 (94990, 110315) | 50379 (46087, 54549) | 51843 (45708, 58032) |
| Deaths per million | 914 (850, 987) | 938 (858, 1016) | 892 (787, 999) |
| % total CMD deaths | 22.9 | 22.3 | 23.5 |
| **High total cholesterol** (> 3.8 mmol/L) |  |  |  |
| CMD deaths | 56699 (41602, 77338) | 29351 (21150, 39788) | 27348 (15950, 44917) |
| Deaths per million^3^ | 507 (372, 692) | 547 (394, 741) | 471 (274, 773) |
| % total CMD deaths^4^ | 12.7 | 13.0 | 12.4 |
| **High fasting plasma glucose** ( > 5.3mmol/L) |  |  |  |
| CMD deaths | 49322 (41370, 58287) | 25738 (20138, 32012) | 23585 (18211, 29780) |
| Deaths per million^3^ | 441 (370, 521) | 479 (375, 596) | 406 (313, 512) |
| % total CMD deaths^4^ | 11.1 | 11.4 | 10.7 |
| **High intake of sodium** (>1000mg/day)^5^ |  |  |  |
| CMD deaths | 57192 (36408, 76101) | 33241 (21310, 44332) | 23951 (15384, 32777) |
| Deaths per million^3^ | 512 (326, 681) | 619 (397, 826) | 412 (265, 564) |
| % total CMD deaths^4^ | 12.8 | 14.7 | 10.9 |
|  |  |  |  |
| ^1^Due to joint distributions, multicausality, interaction, and because the effects of some risk factors are partly mediated through other risk factors, the number of deaths attributable to different risk factors cannot be summed. For example, part of the burden due to high BP is due to high sodium intake and low intakes of fruits and vegetables; whereas much of the burden due to high BMI is mediated by high BP, cholesterol, and glucose. Thus, the numbers of deaths attributable to each risk factor should be considered the total numbers of deaths due to this factor, including its upstream determinants and downstream mediators.  ^2^Total cardiometabolic deaths due to CHD (ICD-10 codes I20–I25), ischemic stroke (I63, I65–I67, I69.3), hemorrhagic/other non-ischemic stroke (I60-62, I69.0-2), hypertensive heart disease (I11–I13), aortic aneurysm (I71), rheumatic heart disease (I01, I02.0, I05–I09), inflammatory heart disease (I33, I42), other CVDs, and diabetes mellitus (E10–E14).  ^3^ Based on the number of people in the same stratum of the population (e.g. men, 25-44y etc);  ^4^ Based on the total number of deaths in the same stratum of the population (e.g. men, 25-44y etc)  ^5^only mediated effects through blood pressure | | | |
